# Jinlian Xiaodu Decoction Protects against Bleomycin-Induced Pulmonary Fibrosis in Rats

**DOI:** 10.1155/2022/4206364

**Published:** 2022-06-23

**Authors:** Zhiqiang Wu, Qin He, Feibao Tao, Xuxing Ye, Saibin Wang, Yijun Zhu, Liang Zhu, Bin Xu

**Affiliations:** ^1^Department of Traditional Chinese Medicine, Affiliated Jinhua Hospital, Zhejiang University School of Medicine, 321000 Jinhua City, Zhejiang Province, China; ^2^Department of Respiratory Medicine, Affiliated Jinhua Hospital, Zhejiang University School of Medicine, 321000 Jinhua City, Zhejiang Province, China; ^3^Department of Clinical Laboratory, Affiliated Jinhua Hospital, Zhejiang University School of Medicine (Jinhua Municipal Central Hospital), 321000 Jinhua City, Zhejiang Province, China

## Abstract

**Background:**

Jinlian Xiaodu Decoction (JXD) was reported to have anti-inflammatory and lung protection effects. This study aimed to explore the role and mechanism of JXD on bleomycin (BLM)-induced pulmonary fibrosis (PF).

**Methods:**

The UHPLC-Q/TOF-MS system was applied to analyze JXD composition. The PF model was established by BLM intratracheal administration in Wistar rats. Subsequently, BLM-treated rats were intragastrically administered with dexamethasone (DXM, 1 g/kg/d) or JXD (3.5, 7 or 14 g/kg/d). Next, the lung coefficient was calculated; H&E, Masson, and TUNEL staining were used for lung morphological analysis and apoptosis assessment. Bronchoalveolar lavage fluid (BALF) biochemical analysis was conducted to count the inflammatory cell number. The expression of inflammatory factors mRNA in the lung tissue and BALF were measured by qRT-PCR. The content and activity of oxidative stress-related proteins were detected. The expression of PF-related, apoptosis-related, and TGF-*β*1 pathway-related protein were assessed by immunohistochemistry or Western blot.

**Results:**

Twenty-six compounds were identified from JXD in both negative and positive ion modes. In BLM-induced rats, JXD reduced the lung coefficient and alleviated PF injury. JXD decreased inflammatory cell count and TNF-*α*, IL-1*β*, IL-6, and MCP-1 content. Meanwhile, JXD blunted BLM-induced oxidative stress and a high level of HYP. Furthermore, TUNEL analysis found that JXD inhibited cell apoptosis and increased Bcl-2/Bax ratio in BLM-induced lung. Moreover, JXD relieved the role of BLM on *α*-SMA, TGF-*β*1, collagen I, fibronectin, E-cadherin protein expression, and the phosphorylation of Smad2/3 in PF rat.

**Conclusion:**

This study revealed the protective effect and possible element of JXD on BLM-caused PF.

## 1. Introduction

Pulmonary fibrosis (PF) is a potentially lethal inflammatory disease, and it has been reported that the incidence of PF is 13 to 14 cases per 1,00,000 persons [1]. Fibroblasts differentiation, myofibroblasts proliferation, honeycomb lesions, and relative abnormality in lung tissue are the common pathological symptoms of PF [2]. Furthermore, the pathogenesis of PF is related to the promotion of *α*-smooth muscle actin (*α*-SMA) expression, the destruction of alveolar-capillary units as well as the overdeposition of extracellular matrix (ECM) [3]. In addition, the fibrous collagen (collagen I, III, V, and VI) leads to the structural remodeling of lung fibrosis [4]. In fact, in addition to lung transplantation, there is no effective treatment for PF now. However, relative to other organ transplantation, patients undergoing lung transplantation have a worse long-term survival (median survival: heart, 10.7 years; liver, 8.5 years; lung, 6.0 years) [5]. Therefore, there is an urgent need to develop new methods for the treatment of PF.

Traditional Chinese medicine (TCM) is widely applied for preventing and treating diseases [6, 7]. They are considered as natural remedies, with advantages such as high efficacy, affordability, minimal or no side effects, and wide variety [8, 9]. Jinlian Xiaodu Decoction (JXD) is based on *Anoectochilus*, *Polygonum cuspidatum*, and Qiliang and made with the addition and subtraction of the card. It has the effects of clearing heat, removing dampness, and detoxifying [10]. Modern pharmacological study has shown that *Anoectochilus* contains flavonoids, alkaloids, steroids, polysaccharides, volatile oils, terpenoids, and other chemical components [11]. *Anoectochilus* has been documented to have multiple biological effects previously, such as antioxidant [12] and liver protection effect [13]. Previous studies have demonstrated that the components of JXD have been used to treat some lung diseases, including chronic obstructive pulmonary disease [14], acute lung injury [15], radiation-induced pulmonary injury [16], and lung cancer [17]. Nevertheless, to date, the effect of JXD on PF remains unknown.

Bleomycin (BLM) is the most commonly used agent in animal PF model [18]. In the repair and fibrosis stage, intratracheal instillation of BLM can cause acute interstitial and macrophage activation, intracellular inflammation, and the upregulation of transforming growth factor-*β* (TGF-*β*) and tumor necrosis factor-*α* (TNF-*α*) [19, 20]. Hence, in this study, we induced an animal PF model by BLM intratracheal administration in Wistar rats to evaluate the role of JXD in BLM-induced PF.

## 2. Materials and Methods

### 2.1. Animals

Male Wistar rats (bodyweight 175–225 g) purchased from Shanghai SLAC Laboratory Animal Co., Ltd. (Certiﬁcate No. SCXK (Hu) 2017-0005; China) were maintained in the condition of specific-pathogen free. Rats were kept in an air-conditioned environment (temperature 22°C; humidity 55%) and supplied with a standard diet and water. All animal experiments were performed with the approval of the Animal Ethics Committee of Hangzhou Eyoung Biomedical Research and Development Center (202011-1301), and the experiments were conducted according to the guidelines of the Chinese Council on Animal Care.

### 2.2. Preparation of JXD Decoction

JXD contains *Anoectochilus* (5 g), Qiliang (20 g), and *Polygonum cuspidatum* (15 g), which were obtained from the same lot in the Department of Pharmacy in the Affiliated Jinhua Hospital, Zhejiang University School of Medicine (Jinhua, China) after identification by a pharmacist. Subsequently, the water decoction was prepared by the traditional decoction method [21]. First, *Anoectochilus*, Qiliang, and *P. cuspidatum* were mixed at a ratio of 1 : 4 : 3 followed by macerating in distilled water for 60 min at room temperature. Then, the mixture was heated to boiling at high heat and maintained boiling at low heat for 30 min. Next, the water decoction was filtered with a 0.22 *μ*m membrane and concentrated to a density of 1, 2, and 4 g/mL. Hereafter, the extract was frozen at −80°C for later use. All experiments were carried out with the same lot of JXD.

### 2.3. Composition Analysis of JXD

To analyze the composition of JXD, 0.25 g of JXD (0.5 g/mL) was taken to dilute with 25 mL of aqueous methanol (50%), vortexed for 1 min, centrifuged 20 min at 14000 r/min, then the supernatant was taken and analyzed by the UHPLC-Q/TOF-MS system. The chromatographic conditions and mass spectrum conditions are given in Table S1. SCIEX OS software was applied for data collecting and processing.

### 2.4. BLM-Induced PF Model and JXD Treatment

All rats were deeply anesthetized by injecting 3% pentobarbital intraperitoneally (50 mg/kg), then 5 mg/kg BLM (dissolved in saline) was injected into the trachea, followed by an immediate rotation of the rats to assure BLM was uniformly distributed in the lung [22]. The rats in the control group were received saline in an identical amount. The rats stimulated by BLM were randomly divided into five groups, BLM, BLM + JXD 3.5 g/kg, BLM + JXD 7 g/kg, BLM + JXD 14 g/kg, and DXM groups, with six rats in each group. Subsequently, rats of the BLM + JXD 3.5 g/kg, BLM + JXD 7 g/kg, and BLM + JXD 14 g/kg groups were intragastrically administered with JXD at a dose of 3.5 g/kg/day (with a concentration of 1 g/mL), 7 g/kg/day (2 g/mL), and 14 g/kg/day (4 g/mL) [23], respectively. The conversion coefficient based on body surface area between human and rats is 6.3 times and the daily dose of JXD for an adult is 40 g. The rats from the DXM group were intragastrically administered with dexamethasone (DXM, 1 g/kg/day), and the rest rats received an equal volume of saline. After treating with JXD for 28 days, the rats were euthanized, their body weight were detected, their lung tissues were harvested, measured and stored for further analysis.

### 2.5. Lung Coefficient

Upon the detection of lung weight and body weight, the lung coefficient was calculated to measure water content in the lung tissue using the following equation: lung wet weight/body weight × 100% [24].

### 2.6. Bronchoalveolar Lavage Fluid (BALF) Biochemical Analysis

At the end of the research, a 20-gage catheter was cannulated to the trachea of the rats. Subsequently, BALF was collected by injecting 1.0 mL of saline into the right lung of the rats and then withdrawing. The injecting process was repeated five times. Next, the BALF was centrifuged for 5 min at 3000 r/min and 4°C, the supernatant was abandoned, and the PBS was added to resuspend the cell pellet. Hereafter, the total cell number was calculated with a hemocytometer by extracting 10 *μ*l of cell suspension. Next, 30 *μ*l of cell suspension was aspirated to prepare a cell smear and stained by Wright-Giemsa (Jiancheng, D010) to analyze the different cell types using an optical microscope.

### 2.7. Histological Analysis

The lung tissues were fixed with paraformaldehyde (4%), embedded in paraffin, sliced into 5 *μ*m-thick section, then deparaffinized in xylene and dehydrated in ethanol. After that, the sections were stained by H&E (MDL, MD911467) and Masson's trichrome (Google Biological, G1006) staining for morphological study in strict accordance with the instructions. In order to evaluate alveolitis and fibrosis, the sections stained with H&E and Masson's trichrome were assessed using a light microscope by a pathologist blinded to the treatment groups.

### 2.8. qRT-PCR Analysis

Total RNA in the lung tissue and BALF of the rats were isolated by RNA extraction reagents (Biyuntian, P0013D). After that, cDNA was amplified from RNA with cDNA reverse-transcription kit (CWBIO, CW2569). The qRT-PCR was performed using SYBR Green qPCR kit (CWBIO, CW2601) and GAPDH was taken as a reference. 2^−ΔΔCt^ method was utilized to calculate the related RNA expression. The list of qRT-PCR primers is given in Table 1.

### 2.9. Measurement of Hydroxyproline (HYP), NO, MDA Content, and SOD, GSH Activity

The lung tissue from the rats were homogenized, and then centrifuged for 10 min at 3500 r/min and 4°C. After that, the lung tissue homogenate was collected to measure HYP content with rat HYP ELISA kit (Meimian, MM-0307R1), MDA, NO content, and GSH, SOD activity by automatic biochemical analyzer (Abbott Laboratories, C16000). All steps were complied with the operation instructions.

### 2.10. Immunohistochemistry

The lung tissues were sliced into 5 *μ*m-thick sections followed by fixation and paraffin embedding. After dewaxing, the sections were covered with 0.01 M citrate buffer (MDL, MD911411) to repair the antigen, treated with 3% H_2_O_2_ for ceasing the activity of endogenous peroxidase, and then incubated with 5% BSA to block unspecific binding. Subsequently, the sections were coincubated overnight with the primary antibodies against collagen I (1 : 200, Affnity, AF7001) and *α*-SMA (1 : 200, Affnity, AF1032) at 4°C. Afterward, the sections were incubated for another 30 min at 37°C with the secondary antibodies. Following rinsing, the sections were stained with DAB (MDL, MD912068) and hematoxylin staining, examined by microscopy. The software of ImageJ was employed to quantify the collagen I and *α*-SMA-positive cells.

### 2.11. Terminal dUTP Nick-End Labeling (TUNEL)

The lung tissue paraffin sections were taken for routine deparaffinization, antigen retrieval, and sealing. Subsequently, incubating the sections with proteinase K solution at room temperature. After washing with PBS, the slices were incubated with TUNEL reaction solution (Roche, 11684817910) at room temperature. Later, the slices were rinsed and further stained with DAPI in the dark. Stained sections were then rinsed and coverslipped. Finally, the image was observed under an inverted fluorescent microscope and analyzed by ImageJ software.

### 2.12. Western Blot Analysis

The lung tissues were treated with RIPA lysate (Beyotime Biotechnology, P0013D) to isolate the total protein. Subsequently, the protein concentration was measured by BCA assay (Solarbio, pc0020). Next, the same amounts of proteins were separated on 10% SDS-PAGE and transferred to PVDF membranes (GE Healthcare Life, pc0020). Following blocking 2 h with 5% skim milk, the membranes were probed with primary antibodies against MMP2 (1 : 1000, AF0577), TIMP-1 (1 : 1000, AF7007), Bax (1 : 1000, AF0120), Bcl-2 (1 : 1000, AF6139), *α*-SMA (1 : 1000, AF1032), TGF-*β*1 (1 : 1000, AF1027), collagen I (1 : 1000, AF7001), fibronectin (1 : 1000, AF5335), E-cadherin (1 : 1000, AF0131), p-Smad2 (1 : 1000, AF8314), Smad2 (1 : 1000, AF6449), p-Smad3 (1 : 1000, AF8315), Smad3 (1 : 1000, bs-3484R), and GAPDH (1 : 5000, AF7021) at 4°C overnight. Then the membranes were rinsed and reincubated with specific secondary antibody for 1 h at 37°C. The films were developed by ECL reagent. The densitometry analysis of the immunoreactive bands was performed by Image J program software. All the primary antibodies were bought from Affinity, except Smad2 from bioss.

### 2.13. Statistical Analysis

The data were presented as mean ± SD and analyzed by SPSS 16.0. One-way ANOVA and SNK tests were applied for multigroup comparison. The Kruskal–Wallis H test was applied, if variances were not equal. *P* < 0.05 was considered a statistically significant difference.

## 3. Results

### 3.1. Composition Analysis of JXD

**Figure 1:**
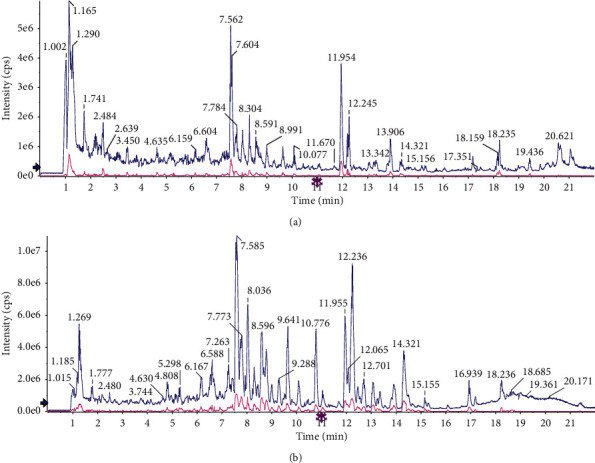
The total ion flow diagram of JXD by UHPLC-Q/TOF-MS in (a) positive ion mode and (b) negative ion mode.

After detecting JXD sample solution with UHPLC-Q/TOF-MS, we obtained the total ion flow chromatogra. The compounds were qualitatively identified by comparison and screening using TCM MS/MS Library of SCIEX OS software.  Figure 1, Tables S2 and S3 reveal the identification results. Twenty-six compounds were identified from JXD in both negative and positive ion modes. Most of these compounds were flavanoids, glycosides, organic acids, amino acids, etc.

### 3.2. JXD Reduced the Lung Coefficient in the PF Rat Model

As given in Table 2, after injecting BLM, relative to the control group, the rats in the BLM group showed a remarkable upregulated lung coefficient (*p* < 0.01), which was reversed by JXD or DXM treatment, and the protective effect of JXD was dosage-dependent (*p* < 0.05).

### 3.3. JXD Reversed BLM-Induced Cell Inflammation

**Figure 2:**
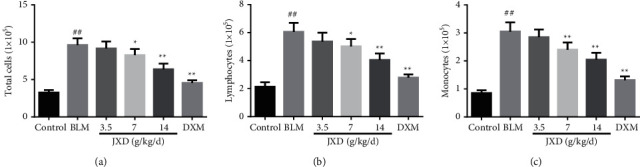
Cell count and classification in BALF of rats. (a) The number of total cells. (b) The number of lymphocytes. (c) The number of monocytes. Data were expressed as mean ± SD. ^##^*P* < 0.01 vs. control. ^*∗*^*P* < 0.05, ^*∗∗*^*p* < 0.01 vs. BLM. *n* = 6.

**Figure 3:**
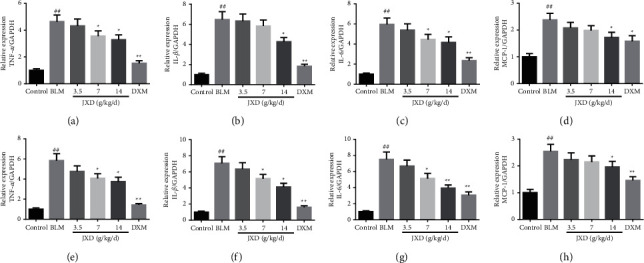
JXD reversed inflammation of the lung tissue and BALF in PF rats. (a–d) qRT-PCR showing that the expression of TNF-*α*, IL-1*β*, IL-6, and MCP-1 mRNA in the lung tissue decreased under the influence of JXD after being induced by BLM. (e–h) qRT-PCR showing that the expression of TNF-*α*, IL-1*β*, IL-6, and MCP-1 mRNA in the BALF decreased under the influence of JXD after being induced by BLM, *n* = 3. Data were expressed as mean ± SD. ^##^*P* < 0.01 vs. control. ^*∗*^*P* < 0.05, ^*∗∗*^*p* < 0.01 vs. BLM.

In order to further evaluate the efficacy of JXD on cell infiltration of PF, we collected BALF and performed cell count (Figure 2). The total cell number was enhanced significantly after the rats were treated with BLM. However, the increased total cell number was significantly reduced upon JXD or DXM treatment. In addition, the number of lymphocytes and monocytes in the BLM group were significantly higher than those in the control group. It was worth noting that upon treating with varying doses of JXD, the counts of monocytes and lymphocytes were significantly reduced in a dose-dependent manner. Meanwhile, qRT-PCR showed that JXD treatment inhibited the expression of TNF-*α*, IL-*β*, IL-6, and MCP-1 mRNA both in lung tissue and BALF of PF rats (Figure 3).

### 3.4. JXD Alleviated BLM-Induced PF Injury

**Figure 4:**
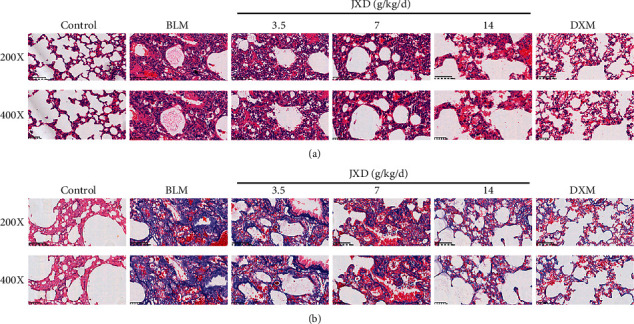
JXD alleviated lung damage caused by BLM. (a) Representative images of H&E staining. (b) Representative images of Masson's trichrome staining. Top panels, ×200 magnification; bottom panels, ×400 magnification.

The effect of JXD on BLM-induced PF was measured by H&E and Masson's trichrome staining. As shown in Figure 4(a), the rats in the control group exhibited normal lung tissue structure, while the rats in the BLM group showed markedly thickened alveolar compartment, disappeared alveolar space, accompanied with a lot of inflammatory cell infiltration. However, the situation was obviously improved in the BLM + JXD 7 g/kg, BLM + JXD 14 g/kg, or DXM group. Meanwhile, as shown in Figure 4(b) by Masson's trichrome staining, massive blue collagenous fiber was found in the lung tissue of the rats in the BLM group. By contrast, less blue collagen was observed in rats treated with BLM or DXM.

### 3.5. Effect of JXD on HYP, NO, MDA, SOD, and GSH in the PF Rat Model

**Figure 5:**
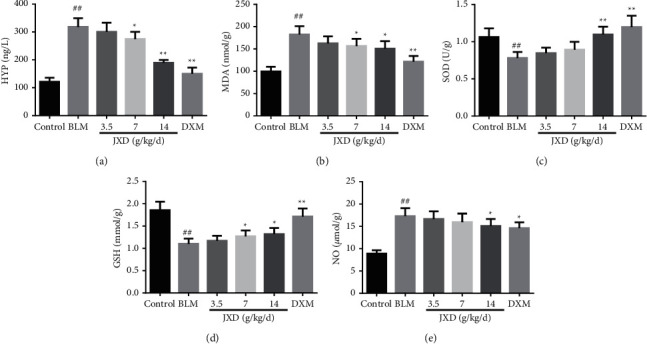
JXD suppressed the BLM-induced oxidative stress. (a–e) The content of hydroxyproline (HYP) (a), MDA (b), and SOD (c), the activity of GSH (d) and NO (e), *n* = 6. Data were expressed as mean ± SD. ^##^*P* < 0.01 vs. control. ^*∗*^*P* < 0.05, ^*∗∗*^*p* < 0.01 vs. BLM.

We assessed the content of HYP to confirm the collagen accumulation in lung tissues (Figure 5(a)). In this test, administration of JXD evidently reversed the high level of HYP in rats induced by BLM in a concentration-dependent manner, which was in agreement with Masson's trichrome staining. Subsequently, to determine if JXD attenuates PF caused by BLM was associated with antioxidant activity, the content of some oxidizing substances were examined, including MDA, NO, SOD, and GSH (Figures 5(b)–5(e)). After treating with BLM, the content of MDA and NO in the lung tissue were notably enhanced (*p* < 0.01), while the activities of SOD and GSH in the lung tissue were obviously reduced (*p* < 0.01). Intriguingly, BLM-induced high content of MDA and NO and decreased activity of SOD and GSH were rescued by treating JXD.

### 3.6. Effect of JXD on Collagen I and *α*-SMA Protein Expression in the PF Rat Model

**Figure 6:**
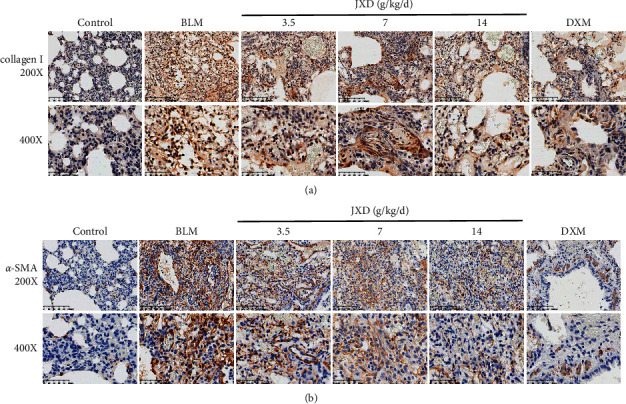
The effect of JXD on the expression of collagen I and *α*-SMA in lung tissue of BLM-induced rats. Top panels, ×200 magnification; bottom panels, ×400 magnification.

The expression level of collagen I and *α*-SMA protein were dramatically elevated in the BLM-treated rats (*p* < 0.01,Figure 6). Nevertheless, after treating with JXD or DXM, the protein expression of collagen I as well as *α*-SMA were reduced; meanwhile, the effect of JXD on downregulating collagen I and *α*-SMA expression were in a dose-dependent manner.

### 3.7. JXD Reversed the BLM-Induced Apoptosis and Related Protein Expression in the PF Rat Model

**Figure 7:**
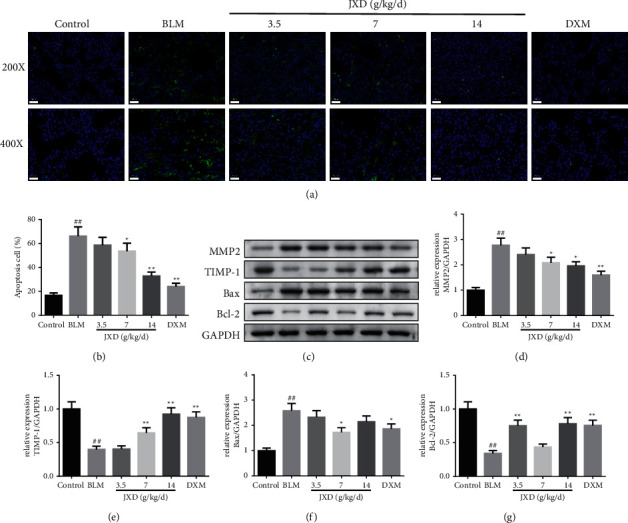
Effect of JXD on the cell apoptosis in pulmonary tissue of BLM-induced PF rats. (a) The apoptotic cells in pulmonary tissues were stained by tunel test kit and observed with a fluorescence microscope (magnification, 200×). (b) The quantitative result of TUNEL staining, *n* = 6. (c) The representative band of Western blot. (d)–(g) The quantitative result of Western blot, *n* = 3. Data were expressed as mean ± SD. ^##^*P* < 0.01 vs. control; ^*∗*^*p* < 0.05, ^*∗∗*^*p* < 0.01 vs. BLM.

TUNEL analysis result of lung tissues revealed the number of apoptotic cells in the BLM group was evidently higher than that of the control group. On the contrary, JXD (7 g/kg, 14 g/kg) or DXM could markedly reduce the number of apoptotic cells (*p* < 0.05, Figures 7(a) and 7(b)). Western blot was conducted to measure the expression level of MMP2, TIMP-1, and apoptosis-associated proteins and found that the addition of JXD decreased the MMP2 and apoptosis protein Bax expression but increased TIMP-1 and antiapoptosis protein Bcl-2 expression.

### 3.8. JXD Relieved the BLM-Induced PF via the TGF-*β*1 Pathway

**Figure 8:**
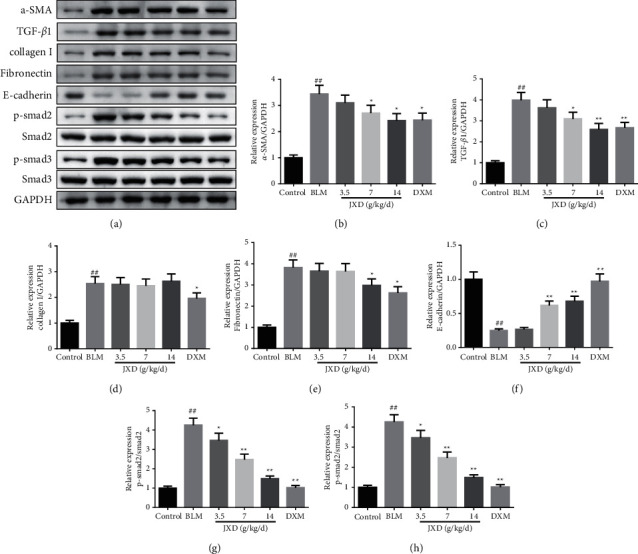
JXD reversed PF by regulating TGF-*β*1 pathway. (a) The representative band of Western blot. (b)–(h) The quantitative result of Western blot. Data were expressed as mean ± SD. ^#^*P* < 0.05/^##^*p* < 0.01 vs. control; ^*∗*^*p* < 0.05/^*∗∗*^*p* < 0.01 vs. the BLM group. *n* = 3.

In order to further clarify the possible molecular mechanism of JXD attenuating BLM-induced PF, the protein expression of *α*-SMA, TGF-*β*1, collagen I, fibronectin, E-cadherin, p-Smad2, Smad2, p-Smad, and Smad3 were measured. Western blot analysis revealed that JXD not only downregulated the expression of *α*-SMA, TGF-*β*1, collagen I, fibronectin protein, and the ratio of p-Smad2/Smad2 and p-Smad3/Smad3 but also upregulated the expression level of E-cadherin protein in the rat PF model (Figure 8).

## 4. Discussion

TCM has been widely used in the treatment of diseases for thousands of years. Various studies have demonstrated that TCM can be used to treat PF and other diseases [25, 26]. For instance, a study has demonstrated that *Poria cocos* Wolf treatment inhibits TGF-*β*1-stimulated renal fibroblast ECM accumulation, fibrosis formation as well as proliferation by suppressing the Smad3, PDGF-C, and MAPK pathway [27]. Furthermore, Zhou et al. have proved that Taohe-Chengqi decoction extract exerts its antirenal fibrosis effect by regulating inflammatory, EMT process as well as ECM deposition [28]. *A. formosanus* is one of the most precious TCMs. *A. formosanus* has a strong antioxidant effect, can strengthen immune function in patients with tumor treatment without toxic side effect [29, 30]. In this study, we detected the JXD sample solution with UHPLC-Q/TOF-MS, and identified 26 compounds in both negative and positive ion mode.

BLM intratracheal administration is the most widely employed experimental model of PF research at present [31]. Delivering BLM to the lung will lead to lung damage, inflammation response, and subsequent fibrosis [32]. In this study, BLM intratracheal administration caused the typical manifestation of PF, such as upregulated lung coefficient, injured lung tissue, increased lymphocytes and monocytes, and increased inflammatory cytokines. These symptoms were all evidently relieved by 28 days of JXD or DXM treatment, and the protective effect of JXD against PF was in a dose-dependent manner. Hence, our findings indicated JXD had a therapeutic effect for BLM-induced PF.

PF is a serious disease generally considered irreversible and has a high mortality [33]. The early manifestation of PF is mainly demonstrated by alveolitis, which then develops into PF during the latter stage [34]. Study has shown that BLM-induced PF is accompanied with oxidative stress [35]. The current understanding of the PF pathogenesis is changing from chronic inflammation to disordered fibroproliferation [36]. In this study, we demonstrated that JXD suppressed cell apoptosis by regulating apoptosis-related protein expression. In addition, JXD alleviated BLM-induced oxidative stress by decreasing the content of NO as well as increasing the activity of GSH, SOD, thus reducing MDA content. Importantly, JXD decreased the level of HYP, which is a unique component of collagen fiber [37]. Thus, it was likely that JXD treatment suppressed cell apoptosis, attenuates oxidative stress, and fibroproliferation during the progress of PF in BLM-induced rats.

MMP, a zinc-dependent endopeptidase, is responsible for the digestion of ECM [38]. Excessive deposition of ECM is one of the characteristics of PF. The degradation of ECM is mainly achieved by MMPs, whereas the degradation efficiency is tightly controlled by TIMPs [16]. Research has proved that the upregulation of MMPs expression in PF can break out the balance of MMPs/TIMPs, which can be a major mechanism related to PF [39]. Previous studies have demonstrated that MMP3 and MMP7 promote PF by downregulation of antifibrotic mediators, upregulation of profibrotic mediators, and promotion of cell migration in animal models [40]. The primary function of TIMP-1 is to suppress MMPs activity [41]. In the study, we measured TIMP-1 and MMP2 expression, and found that MMP2 expression was significantly upregulated, while TIMP-1 expression was dramatically decreased after rats were treated with BLM. Meanwhile, JXD or DXM treatment could notably decrease the MMP2 expression and increase the TIMP-1 expression. These results clearly suggested that the protective effect of JXD against BLM-induced PF was related to the decreased MMP2 and the increased TIMP-1.

Epithelial-mesenchymal transition (EMT), a critical process of the occurrence and progression of PF, is closely related to damage repair, tissue regeneration, and organ fibrosis [42]. TGF-*β* pathway is a main inducer of the EMT progression, which can induce *α*-SMA expression and stimulate collagen I synthesis [43]. *α*-SMA can repress the epithelial gene expression and act as an EMT-activator [44]. The main feature of PF is the deposition of the ECM, collagen fiber is the main component of ECM, and collagen I is vital in the synthesis of collagen [45]. The main characteristics of EMT are the blunted cell adhesion molecule (such as E-cadherin) expression as well as the elevated extracellular matrix protein (such as fibronectin) expression [46]. In this study, intratracheal administration of BLM led to the upregulation of TGF-*β*, *α*-SMA, collagen I, and fibronectin expression, and the downregulation of E-cadherin expression in the lung tissues. However, JXD therapy suppressed BLM-induced upregulation of TGF-*β*, *α*-SMA, collagen I, fibronectin, and promoted BLM-induced downregulation of E-cadherin, which indicated that JXD may reduce BLM-induced PF via inhibiting TGF-*β*1 pathway.

However, there are some limitations in our study. First, we did not perform a time-dependent study. Furthermore, based on the development of fibrosis upon BLM treatment, we should treat rats with JXD from 15th day upon BLM administration. In the future, we will conduct time-dependent study and supplied rats with JXD at day 15 up on BLM instillation to better verify the results.

In conclusion, our data demonstrate that JXD was effective in alleviating BLM-induced PF via TGF-*β* pathway. Therefore, JXD therapy, as an adjunctive therapy for PF patients, has broad therapeutic potential and is worth investigating and evaluating in further study.

## Figures and Tables

**Table 1:** qRT-PCR primers.

	Primer	Reverser
Rat TNF-*α*	ATCCGAGATGTGGAACTGGC	CGATCACCCCGAAGTTCAGT
Rat IL-6	CACTTCACAAGTCGGAGGCT	CACTTCACAAGTCGGAGGCT
Rat IL-1*β*	CCTTGTCGAGAATGGGCAGT	CAGGGAGGGAAACACACGTT
Rat MCP-1	CTGAGTTGACTCCTACTGTGGA	TCTTCCCAGGGTCGATAAAGT
Rat GAPDH	GATGGTGAAGGTCGGTGTGA	TGAACTTGCCGTGGGTAGAG

**Table 2:** Lung coefficients in the PF rat model (mean ± SD, *n* = 6).

Group	Lung coefficients (%)
Control	0.91 ± 0.07
BLM	1.47 ± 0.15^##^
BLM + JXD 3.5 g/kg	1.42 ± 0.13
BLM + JXD 7 g/kg	1.29 ± 0.12^*∗*^
BLM + JXD 14 g/kg	1.24 ± 0.13^*∗*^
DXM	1.12 ± 0.10^*∗∗*^

Note: *n* = 6; values were the mean ± SD;^##^*p* < 0.01 vs. control. ^*∗*^*p* < 0.05, ^*∗∗*^*p* < 0.01 vs. BLM.

## Data Availability

The data used to support the findings of this study are included within the article.

## Supplementary Materials

Supplementary Materials Table S1: chromatographic conditions and mass spectrum conditions. Table S2: high-resolution mass spectrometry data and elemental composition of AFTDD (positive ion mode). Table S3: high-resolution mass spectrometry data and elemental composition of AFTDD (negative ion mode).
