# Supplementary material for: Jinlian Xiaodu Decoction Protects against Bleomycin-Induced Pulmonary Fibrosis in Rats
Source: Evid Based Complement Alternat Med. 2022 Jun 23;2022:4206364. doi: 10.1155/2022/4206364 (PMC9246571; doi:10.1155/2022/4206364)
Supplement: Supplementary Materials — Table S1: chromatographic conditions and mass spectrum conditions. Table S2: high-resolution mass spectrometry data and elemental composition of AFTDD (positive ion mode). Table S3: high-resolution mass spectrometry data and elemental composition of AFTDD (negative ion mode). [file 4206364.f1.docx]

**Table S1 Chromatographic conditions and mass spectrum conditions**

| Column type | ACQUITY UPLC BEH C18 column, 150 × 2.1 mm, 1.7 μm |
| --- | --- |
| Flow rate | 0.3 mL/min |
| A phase | 0.1% formic acid acetonitrile |
| B phase | 0.1% formic acid solution |
| (%B), time | 99-80%, 0-6 min |
| (%B), time | 80-60%, 6-15min |
| (%B), time | 60-15%, 15-18 min |
| (%B), time | 15-1% , 18-20 min |
| (%B), time | 1%, 20-22 min |
| Column temperature | 40 °C |
| Injection volume | 3 μL |
| Ion Source Gas1 | 55 |
| Ion Source Gas2 | 45 |
| Curtain gas | 35 |
| Source temperature | 600 °C |
| IonSapary Voltage Floating | 5500 V/-4500 V(positive and negative ion mode) |
| TOF-MS scan m/z range | 50-1500 Da |
| Production scan m/z range | 25-1500 Da |
| TOF-MS scan accumulation time | 0.25 s/spectra |
| Product ion scan accumulation time | 0.035 s/spectra |
| Declustering potential | +60 V (positive and negative ion mode) |
| Collision Energy | 35±15 eV |
| Exclude isotopes | within 4 Da |
| Candidate ions to monitor per cycle | 12 |

**Table S2 High resolution mass spectrometry data and elemental composition of AFTDD (Positive Ion Mode).**

| No. | Component Name | Area | Retention Time | Formula | Precursor Mass | Found At Mass | Mass Error (ppm) | Library Score | Isotope Ratio Difference |
| --- | --- | --- | --- | --- | --- | --- | --- | --- | --- |
| 1 | L(+)-Arginine | 12610000 | 1.14 | C6H14N4O2 | 175.119 | 175.1187 | -1.3 | 85.7 | 2.7 |
| 2 | Trigonelline | 1137000 | 1.21 | C7H7NO2 | 138.055 | 138.0551 | 0.9 | 96.8 | 3 |
| 3 | Proline | 991900 | 1.24 | C5H9NO2 | 116.071 | 116.0709 | 2.3 | 98.5 | 1.5 |
| 4 | Adenine | 625700 | 1.68 | C5H5N5 | 136.062 | 136.0617 | -0.8 | 99.1 | 4.4 |
| 5 | Cytidine | 42080 | 1.73 | C9H13N3O5 | 244.093 | 244.0935 | 2.9 | 100 | 3.1 |
| 6 | Nicotinic acid | 177800 | 1.74 | C6H5NO2 | 124.039 | 124.0396 | 2.3 | 98.7 | 2.1 |
| 7 | Nicotinamide | 419600 | 1.83 | C6H6N2O | 123.055 | 123.0553 | 0.3 | 96.8 | 4.1 |
| 8 | Adenosine | 2807000 | 2.5 | C10H13N5O4 | 268.104 | 268.1038 | -1 | 100 | 3.5 |
| 9 | Guanosine | 241800 | 2.64 | C10H13N5O5 | 284.099 | 284.0994 | 1.6 | 99.1 | 5.2 |
| 10 | Cinnamic acid | 24390 | 3.45 | C9H8O2 | 149.06 | 149.0601 | 2.9 | 83.7 | 2 |
| 11 | Phenylalanine | 916400 | 3.45 | C9H11NO2 | 166.086 | 166.0863 | 0.3 | 98.9 | 2.4 |
| 12 | 5-Hydroxymethylfurfural | 84340 | 3.56 | C6H6O3 | 127.039 | 127.0391 | 1.2 | 93 | 2 |
| 13 | Epicatechin | 1492000 | 5.29 | C15H14O6 | 291.086 | 291.0865 | 0.5 | 92.6 | 6.5 |
| 14 | Loganic acid | 35690 | 6.15 | C16H24O10 | 377.144 | 377.1458 | 4.2 | 84.1 | 3.2 |
| 15 | Piceatannol | 647300 | 6.51 | C14H12O4 | 245.081 | 245.0806 | -0.9 | 91.6 | 6.4 |
| 16 | Syringaldehyde | 12890 | 7.51 | C9H10O4 | 183.065 | 183.0655 | 1.9 | 87.8 | 3.5 |
| 17 | Resveratrol | 18790000 | 7.57 | C14H12O3 | 229.086 | 229.0858 | -0.6 | 92.1 | 3.4 |
| 18 | Polydatin | 2693000 | 7.58 | C20H22O8 | 391.139 | 391.139 | 0.7 | 94.6 | 6.1 |
| 19 | Isoquercitrin | 290200 | 7.72 | C21H20O12 | 465.103 | 465.1033 | 1.1 | 99.6 | 8.5 |
| 20 | Dihydroquercetin | 3085000 | 8.03 | C15H12O7 | 305.066 | 305.0654 | -0.6 | 95.2 | 6.9 |
| 21 | Isorhamnetin | 682700 | 8.31 | C16H12O7 | 317.066 | 317.0656 | 0.2 | 92 | 3 |
| 22 | Engeletin | 36210 | 8.89 | C21H22O10 | 435.129 | 435.1291 | 1.1 | 81.9 | 6.9 |
| 23 | Paeonol | 24470 | 9.73 | C9H10O3 | 167.07 | 167.0704 | 0.8 | 71.4 | 3.3 |
| 24 | Nodakenin | 3602000 | 11.95 | C20H24O9 | 409.149 | 409.1493 | 0 | 88.5 | 9.8 |
| 25 | Ononin | 135500 | 11.96 | C22H22O9 | 431.134 | 431.1327 | -2.2 | 96.4 | 4.7 |
| 26 | Emodin | 8796000 | 12.25 | C15H10O5 | 271.06 | 271.0597 | -1.6 | 99.4 | 5.5 |

**Table S3 High resolution mass spectrometry data and elemental composition of AFTDD (Negative Ion Mode).**

| No. | Component Name | Area | Retention Time | Formula | Precursor Mass | Found At Mass | Mass Error (ppm) | Library Score | Isotope Ratio Difference |
| --- | --- | --- | --- | --- | --- | --- | --- | --- | --- |
| 1 | Histidine | 29950 | 1.1 | C6H9N3O2 | 154.062 | 154.0621 | -1 | 89.2 | 1.7 |
| 2 | L(+)-Arginine | 507700 | 1.11 | C6H14N4O2 | 173.104 | 173.1043 | -0.5 | 99.2 | 4.1 |
| 3 | Aspartic acid | 145800 | 1.12 | C4H7NO4 | 132.03 | 132.0302 | -0.1 | 91.2 | 0.9 |
| 4 | Glutamic acid | 49120 | 1.14 | C5H9NO4 | 146.046 | 146.0459 | -0.2 | 98.9 | 1.9 |
| 5 | D-(+)-Mannose | 666000 | 1.19 | C6H12O6 | 179.056 | 179.056 | -0.4 | 91.4 | 0.8 |
| 6 | Vitamin C | 38680 | 1.43 | C6H8O6 | 175.025 | 175.0248 | 0.1 | 91.6 | 0.8 |
| 7 | Citric acid | 4944000 | 1.79 | C6H8O7 | 191.02 | 191.0198 | 0.6 | 98.4 | 3.7 |
| 8 | Amber Acid | 135500 | 2.4 | C4H6O4 | 117.019 | 117.0194 | 0.5 | 98.2 | 2.1 |
| 9 | Isoleucine | 40300 | 2.61 | C6H13NO2 | 130.087 | 130.0874 | 0.5 | 100 | 2.5 |
| 10 | Gallic acid | 790400 | 2.79 | C7H6O5 | 169.014 | 169.0142 | -0.4 | 82.6 | 4.4 |
| 11 | Protocatechuic acid | 86060 | 4.05 | C7H6O4 | 153.019 | 153.0192 | -0.7 | 95.5 | 1.6 |
| 12 | Protocatechuic Aldehyde | 187900 | 5.1 | C7H6O3 | 137.024 | 137.0242 | -1.8 | 97.2 | 3 |
| 13 | Mulberroside A | 12960 | 5.85 | C26H32O14 | 567.172 | 567.171 | -1.6 | 82.9 | 3.8 |
| 14 | p-Coumaric acid | 983400 | 7.55 | C9H8O3 | 163.04 | 163.0398 | -1.6 | 97.2 | 3.4 |
| 15 | Polydatin | 32820000 | 7.59 | C20H22O8 | 389.124 | 389.1237 | -1.2 | 98.3 | 9 |
| 16 | Isoquercitrin | 356200 | 7.71 | C21H20O12 | 463.088 | 463.0878 | -0.9 | 100 | 4.8 |
| 17 | Astilbin | 18330000 | 8.04 | C21H22O11 | 449.109 | 449.1085 | -0.9 | 88.3 | 6.2 |
| 18 | Piceatannol | 365900 | 8.39 | C14H12O4 | 243.066 | 243.0662 | -0.5 | 88.4 | 0.9 |
| 19 | Engeletin | 1268000 | 8.98 | C21H22O10 | 433.114 | 433.1137 | -0.8 | 97.5 | 9.9 |
| 20 | Scutellarin | 12620 | 10.06 | C21H18O12 | 461.073 | 461.0717 | -1.8 | 97.3 | 7.8 |
| 21 | Quercetin | 489200 | 11.37 | C15H10O7 | 301.035 | 301.035 | -1.2 | 97 | 8.5 |
| 22 | Emodin-8-glucoside | 33840000 | 12.24 | C21H20O10 | 431.098 | 431.0979 | -1.1 | 91.2 | 8.4 |
| 23 | Naringenin | 289600 | 13 | C15H12O5 | 271.061 | 271.0606 | -2.2 | 97.3 | 7.8 |
| 24 | Trifolirhizin +HCOOH | 333600 | 13.91 | C22H22O10.HCOOH | 491.119 | 491.1181 | -2.8 | 77.2 | 3.6 |
| 25 | Physcion | 2817000 | 13.91 | C16H12O5 | 283.061 | 283.0605 | -2.5 | 99.6 | 8.5 |
| 26 | Apigenin | 9789000 | 18.24 | C15H10O5 | 269.046 | 269.0449 | -2.3 | 97.9 | 8.7 |
